# Effects of imidacloprid and thiamethoxam on the development and reproduction of the soybean aphid *Aphis glycines*

**DOI:** 10.1371/journal.pone.0250311

**Published:** 2021-09-16

**Authors:** Aonan Zhang, Lin Zhu, Zhenghao Shi, Tianying Liu, Lanlan Han, Kuijun Zhao

**Affiliations:** College of Agriculture, Northeast Agricultural University, Harbin, Heilongjiang, PR China; Institut Sophia Agrobiotech, FRANCE

## Abstract

The soybean aphid *Aphis glycines* Matsumura (Hemiptera: Aphididae) is a primary pest of soybeans and poses a serious threat to soybean production. Our studies were conducted to understand the effects of different concentrations of insecticides (imidacloprid and thiamethoxam) on *A*. *glycines* and provided critical information for its effective management. Here, we found that the mean generation time and adult and total pre-nymphiposition periods of the LC_50_ imidacloprid- and thiamethoxam-treatment groups were significantly longer than those of the control group, although the adult pre-nymphiposition period in LC_30_ imidacloprid and thiamethoxam treatment groups was significantly shorter than that of the control group. Additionally, the mean fecundity per female adult, net reproductive rate, intrinsic rate of increase, and finite rate of increase of the LC_30_ imidacloprid-treatment group were significantly lower than those of the control group and higher than those of the LC_50_ imidacloprid-treatment group (*P* < 0.05). Moreover, both insecticides exerted stress effects on *A*. *glycines*, and specimens treated with the two insecticides at the LC_50_ showed a significant decrease in their growth rates relative to those treated with the insecticides at LC_30_. These results provide a reference for exploring the effects of imidacloprid and thiamethoxam on *A*. *glycines* population dynamics in the field and offer insight to agricultural producers on the potential of low-lethal concentrations of insecticides to stimulate insect reproduction during insecticide application.

## Introduction

The soybean aphid *Aphis glycines* Matsumura (Hemiptera: Aphididae) is native to Asia and mainly distributed in soybean-growing areas in the Far East [1]. It is a common pest of soybeans in North China, as well as in Northeast, North, South, Southwest, Inner Mongolia, and the Ningxia autonomous regions in China [2]. The soybean aphid was first detected in North America in 2000 and has rapidly spread to the north-central and northeastern United States, with recent reports of soybean aphids also in Canada [3]. As a potentially harmful agricultural pest in soybean production, the distribution area and damage scope of soybean aphids have increased annually. Although damage from *A*. *glycines* to soybeans is rarely devastating in Asia, it is considered a primary pest in North America [3,4].

Neonicotinoids exert an excellent control effect on *A*. *glycines* [5,6]. Farmers typically treat soybean seeds prophylactically with neonicotinoids, such as thiamethoxam, and later with foliar sprays, such as imidacloprid and pyrethroids, for aphid control [7,8]. By the late 1990s, neonicotinoids were introduced worldwide owing to their high efficacy against target pests, low toxicity to mammals and other non-target organisms, and wide application range [9].

Neonicotinoid concentrations have gradually decreased from their initial application level due to the interaction of plants, animals, fungi, and bacteria. As a result, some aphids are exposed to low or sublethal concentrations of neonicotinoids below the recommended concentration [10]. Different concentrations of neonicotinoids have different hormesis effects on pests [11]. Ullah et al. [12] used different concentrations of thiamethoxam to induce stress in *Aphis gossypii*, finding that the LC_50_ of thiamethoxam was highly toxic to adults. Treatment of the F0 population of *A*. *gossypii* with LC_15_ thiamethoxam affected the pre-adult stage, longevity, and fertility of the F1 population; however, no significant response was observed at LC_5_, revealing that low-concentration stimulation after exposure to insecticides was the key to pest resurgence [12]. Therefore, it is important to study the hormesis effects of low concentrations of insecticides and their physiological and behavioral effects on pests [13–15], as these effects might drive habitat changes in pest populations, induce the development of resistance, and lead to secondary pest outbreaks and rapid deterioration of the ecological environment [16–19].

In this study, we used the age-stage, two-sex life table to assess the effects of insecticides on pest-population characteristics. By analyzing the survival, development, and reproduction of populations, this allows a comprehensive assessment of the effects of insecticides on the biological fitness of pests and the timing of their progeny resurgence [20–22]. Moreover, it enables improved modeling of the effects of stage-specific mortality, with a greater focus on population-stage-specific differentiation, overlap, and pre-adult mortality relative to traditional approaches [23,24]. It is important to analyze population dynamics and develop effective control programs, and this study provides a reference for the effective application of these two insecticides to control soybean aphids [24].

## Materials and methods

### Laboratory aphid population

The laboratory strain of *A*. *glycines* used in this study was originally collected from a soybean field in Harbin, Heilongjiang Province, China. This strain had been cultured in the laboratory for several years and never exposed to any insecticides. Dongnong 52 soybean plants were used to maintain the *A*. *glycines* strain at Northeast Agricultural University, China. Soybean plants were grown in pots (15 cm diameter × 17 cm depth), with six plants per pot, at 25 ± 1°C with 65% to 70% relative humidity and a 14-:10-h light:dark photoperiod. The laboratory aphid colony was maintained under the same environmental conditions as in the chamber used for plant germination. Twelve pots with soybean plants were placed in a large tray (70cm × 60 cm; L × W) and twice weekly, one-third of the old aphid-infested soybean plants (i.e., the four oldest pots with an aphid infestation) were removed and replaced with new aphid-free plants. Aphids were transferred by placing infested leaves on uninfested plants in order to prevent the accumulation of excessive honeydew and sooty mold and ensure the provision of a homogeneous soybean plant on which the aphids could feed [25].

### Chemical agents

Water-dispersible granules of insecticides [70% imidacloprid (trade name Yashijing) and 50% thiamethoxam (trade name Aketai)] were purchased from North China Pharmaceutical Group Corporation (Hebei, China) and Shaanxi Thompson Biotechnology Co., Ltd. (Shaanxi, China), respectively. Calcium nitrate, potassium nitrate, potassium dihydrogen phosphate, magnesium sulfate, disodium ethylenediaminetetraacetic acid (EDTA), and streptomycin sulfate were purchased from Shanghai Alighting Biochemical Technology Co., Ltd. (Shanghai, China).

### Preparation of culture medium

Non-toxic, transparent plastic Petri dishes (6 cm diameter × 1.5 cm height) were used to perform the bioassay on the first instar nymphs of *A*. *glycines* for the life table study. The components of the plant-nutrient solution concentrate used to prepare the medium were as follows: calcium nitrate (4.1 g), potassium nitrate (2.5 g), potassium dihydrogen phosphate (0.7 g), magnesium sulfate (0.6 g), 1.54% disodium EDTA aqueous solution (5.0 mL), 1×10^6^ U streptomycin sulfate (0.05 g), and distilled water (5.0 L). The diluent was obtained by mixing the plant-nutrient solution concentrate with distilled water (1:3, v/v). The preliminary experiment showed that medium mixed with the diluent of plant-nutrient solution and agar had a better effect on the leaves than that of a solution of distilled water and agar only. Agar was prepared by mixing 1% (w/w) agar powder with diluent and boiling while constantly mixing. After cooling for ~10 min, the warm agar was poured into Petri dishes to a depth of at least 3 mm to 4 mm. At least a 10-mm distance was allowed between the top of the agar and the rim of the Petri dishes. A sharpened metal tube was used to cut leaf discs from clean, untreated leaves, with the diameter of the leaf discs 2-mm less than that of the Petri dishes. The leaf discs were attached to the agar medium with the top-side facing down. The metal tube was sharpened and cleaned regularly to ensure the clean cutting of the leaf discs. *A*. *glycines* on the leaf discs fed on the bottom surface, and each Petri dish was then placed upside down to maintain *A*. *glycines* in a natural feeding state. The incision was maintained to avoid excessive crushing of the tissue at the edge of the leaves while cutting in order to prevent the leaves from rapidly developing mildew.

### Concentration-response bioassay

Concentration-response bioassays were conducted with the first instar *A*. *glycines* nymphs using the leaf-dip method recommended by the Insecticide Resistance Action Committee (http://www.irac-online.org/resources/methods.asp). Insecticidal stock solutions were prepared in 1% acetone and further diluted to different concentrations using distilled water containing 0.05% (v/v) Triton X-100 before use in the concentration-response bioassay. According to preliminary bioassay results, we prepared seven concentrations of imidacloprid (19.95, 13.70, 9.10, 6.10, 3.47, 2.35, and 1.88 mg a.i./L) and thiamethoxam (29.95, 24.98, 14.97, 10.05, 4.94, 3.64, and 1.98 mg a.i./L). Fresh soybean-leaf discs were immersed in the insecticide solutions, with each leaf disc immersed for 10 s, removed from the solution, and placed on paper towels (abaxial surface facing up) to air dry. The control leaf disc was immersed in a solution of distilled water containing 0.05% (v/v) Triton X-100 and 1% acetone. A small drop of distilled water was placed on the surface of the agar prior to laying the leaf on the surface to help the leaf stick to the agar surface. The air-dried leaf discs were attached to the agar medium with the top-side facing down, and the first instar nymphs were then placed on the discs. Treatment details (insecticide, concentration, and date) were recorded for each Petri dish. Sixty first instar nymphs were used for the concentration-response bioassays with insecticides at each concentration, with three replicates used for each concentration and each replicate involving 20 first instar nymphs. Mortality was determined after 24 h of exposure. The first instar nymphs were considered dead if they were found upside down and not moving or if they did not move when prodded with a small paint brush [26]. The toxicity of imidacloprid and thiamethoxam to nymphs was statistically analyzed using the concentration–mortality regression line and the log-probit model of SPSS (v.23.0; IBM Corp., Armonk, NY, USA), and LC_50_ and LC_30_ values were obtained.

### Life table study

Apterous adults (n = 150) were transferred onto 15 leaf discs using a small paint brush, and 10 apterous adults were placed on each leaf disc. Each Petri dish containing a leaf disc was sealed with a close-fitting, ventilated lid. The first instar nymphs were selected 24 h after emergence and placed on a leaf disc pre-impregnated with LC_30_ and LC_50_ imidacloprid and thiamethoxam or a leaf disc pre-impregnated with distilled water containing 0.05% (v/v) Triton X-100 and 1% acetone, with one first instar nymph placed on each leaf disc. Each treated individual was cultured separately as one replicate from the first instar nymph stage to death. Each treatment comprised 100 replicates. The growth, survival, and mortality of individuals were observed and recorded every 24 h. New first instar nymphs were removed after recording to avoid duplicate recording.

### Life table analysis

The age-stage-specific survival rate (*s*_*xj*_, *x* = age, *j* = stage), age-specific survival rate (*l*_*x*_), age-stage-specific fecundity (*f*_*xj*_), and age-specific fecundity (*m*_*x*_) were calculated as previously described [23] and using the following equations:

$$s_{xj}=\frac{n_{xj}}{n_{01}},$$ (1)

$$l_{x}=\sum_{j=1}^{k}s_{xj},$$ (2)

$$m_{x}=\frac{\sum_{j=1}^{k}s_{xj}f_{xj}}{\sum_{j=1}^{k}s_{xj}},$$ (3)

where *n*_01_ represents the number of first instar nymphs, and *k* is the number of stages. The net reproductive rate (*R*_0_), intrinsic rate of increase (*r*), finite rate of increase (*λ*), and mean generation time (*T*) were calculated as previously described [27] and using the following equations:

$$R_{0}=\sum_{x=0}^{\infty }l_{x}m_{x},$$ (4)

$$\sum_{x=0}^{\infty }e^{-r(x+1)}l_{x}m_{x}=1,$$ (5)

$$\lambda =e^{r},$$ (6)

$$T=\frac{\ln\;R_{0}}{r}.$$ (7)

The life expectancy (*e*_*xj*_; i.e., the time that an individual of age *x* and stage *j* is expected to live) was calculated as previously described [28] as:

$$e_{xj}=\sum_{i=x}^{\infty }\sum_{y=j}^{k}s_{iy}^{\prime },$$ (8)

where, *s′*_*iy*_ is the probability that an individual of age *x* and stage *j* would survive to age *i* and stage *y*. Tuan [29] defined the reproductive value (*v*_*xj*_) as the contribution of individuals of age *x* and stage *j* to the future population, with this calculated as:

$$v_{xj}=\frac{e^{r(x+1)}}{s_{xj}}\sum_{i=x}^{\infty }e^{-r(i+1)}\sum_{y=j}^{k}s_{iy}^{\prime }f_{iy}.$$ (9)

The bootstrap technique of TWOSEX-MSChart software [30] was used to replicate samples 100,000 times to estimate the mean value and standard error of the population parameters, mean longevity of the first to fourth instar nymphs and adults, adult and total pre-nymphiposition period, and mean fecundity per female adult. A paired bootstrap test, which is based on the percentile of differences and the 95% confidence interval of a normalized distribution of differences, was used to compare differences among treatments [30]. All curve graphs were generated using SigmaPlot (v.12.0; Systat Software, Inc., San Jose, CA, USA).

## Results

### Concentration-response bioassay with the first instar nymphs of *A*. *glycines*

The LC_50_ and LC_30_ values for imidacloprid and thiamethoxam are listed in Table 1.

**Table 1:** Toxic effects of imidacloprid and thiamethoxam on the first instar nymphs of *A*. *glycines*.

Insecticide	Concentration (mg a.i./L) (95% CI)^−1^		Slope ± SE	χ^2^ (df)
	LC50	LC_30_		
Imidacloprid	4.440 (3.672–5.335)	3.114 (2.425–3.757)	3.402 ± 0.469	0.464 (5)
Thiamethoxam	7.049 (5.394–8.998)	4.184 (2.850–5.460)	2.314 ± 0.339	0.502 (5)

CI, confidence interval; SE = standard error.

### Life history traits

Imidacloprid and thiamethoxam affected the development time, longevity, and fecundity of *A*. *glycines* (Table 2). The development time of the first instar under LC_50_ imidacloprid (F = 38.188, df = 144.337, *P* = 0.000) and thiamethoxam (F = 23.955, df = 151.319, *P* = 0.000) treatments was 0.53-fold and 0.28-fold higher than that of the control, respectively. Additionally, the adult pre-nymphiposition period (APNP) was 4.55- (F = 52.047, df = 122.217, *P* = 0.000) and 4.35-fold (F = 48.657, df = 130.359, *P* = 0.000) higher than that of the control, and the total pre-nymphiposition period (TPNP) was 0.43- (F = 12.446, df = 177.133, *P* = 0.000) and 0.27-fold (F = 6.584, df = 186.558, *P* = 0.000) higher than that of the control under LC_50_ imidacloprid and thiamethoxam treatments, respectively. The fecundity of soybean aphids treated with LC_50_ imidacloprid (F = 1.021, df = 198, *P* = 0.000) and thiamethoxam (F = 0.330, df = 198, *P* = 0.000) decreased by 67.33% and 62.30% relative to the control, whereas adult longevity decreased by 23.05% (F = 51.311, df = 129.068, *P* = 0.000) and 21.78% (F = 38.331, df = 146.438, *P* = 0.000) relative to that of the control, respectively. The development time of the second instar nymphs significantly increased by 19.17% when exposed to LC_50_ imidacloprid (F = 2.169, df = 198, *P* = 0.000), although no significant change was observed in nymphs exposed to LC_50_ thiamethoxam (F = 0.030, df = 198, *P* = 0.055) relative to the control group. However, the APNP in the LC_30_ imidacloprid (F = 9.852, df = 182.661, *P* = 0.000) and thiamethoxam (F = 4.484, df = 191.829, *P* = 0.000) -treatment groups significantly decreased by 75% and 90% relative to the control group, respectively, whereas the fecundity decreased by 50.79% (F = 4.755, df = 188.783, *P* = 0.000) and 46.67% (F = 1.007, df = 198, *P* = 0.000) relative to the control group, respectively. The longevity of adults exposed to LC_30_ imidacloprid (F = 13.707, df = 177.105, *P* = 0.212) and thiamethoxam (F = 0.057, df = 198, *P* = 0.000) significantly decreased by 18.78% and 15.52% relative to that of the control group, respectively. Furthermore, the TPNP of *A*. *glycines* exposed to LC_30_ imidacloprid (F = 18.341, df = 156.090, *P* = 0.000) showed no significant difference relative to that of the control group, whereas those exposed to LC_30_ thiamethoxam (F = 2.789, df = 198, *P* = 0.000) significantly decreased by 7.94%.

**Table 2:** Mean value (± SE) of life history parameters of *A*. *glycines* exposed to imidacloprid and thiamethoxam.

Stages			LC_30_				LC_50_			
	n	Control	n	Imidacloprid	n	Thiamethoxam	n	Imidacloprid	n	Thiamethoxam
L1 (day)	100	1.23 ± 0.04c	64	1.19 ± 0.05c	72	1.14 ± 0.04c	49	1.88 ± 0.09a	53	1.57 ± 0.11b
L2 (day)	93	1.2 ± 0.04b	60	1.17 ± 0.05b	69	1.14 ± 0.04b	44	1.43 ± 0.08a	50	1.28 ± 0.07ab
L3 (day)	90	1.13 ± 0.05a	57	1.14 ± 0.05a	62	1.15 ± 0.05a	41	1.17 ± 0.06a	48	1.15 ± 0.05a
L4 (day)	86	1.01 ± 0.01a	57	1.02 ± 0.02a	61	1.02 ± 0.02a	40	1.1 ± 0.05a	48	1.06 ± 0.04a
Mean longevity of female adult (day)	86	11.02 ± 0.55a	57	8.95 ± 0.56b	61	9.31 ± 0.62b	40	8.48 ± 0.64b	48	8.62 ± 0.55b
APNP (day)	86	0.20 ± 0.04b	55	0.05 ± 0.03c	58	0.02 ± 0.02c	38	1.11 ± 0.15a	43	1.07 ± 0.15a
TPNP (day)	86	4.66 ± 0.07c	55	4.55 ± 0.08c	58	4.29 ± 0.07d	38	6.68 ± 0.20a	43	5.93 ± 0.18b
Fecundity	86	42.49 ± 1.83a	55	20.91 ± 1.39b	58	22.66 ± 1.60b	38	13.88 ± 1.56c	43	16.02 ± 1.37c

Means (±SE) followed by different letters in the same row are significantly different as calculated using the paired bootstrap test at the *P* < 0.05 level. Leaves treated with distilled water were used as the control.

SE = standard error; L1 = mean longevity of the first instar nymphs; L2 = mean longevity of the second instar nymphs; L3 = mean longevity of the third instar nymphs; L4 = mean longevity of the fourth instar nymphs; Fecundity = mean fecundity per female adult.

### Life table and fertility parameters

The *R*_0_, *r*, and *λ* of soybean aphids under LC_50_ imidacloprid and thiamethoxam treatments were significantly lower than those of aphids under LC_30_ treatment and controls. In the LC_50_ imidacloprid- and thiamethoxam-treatment groups, the *R*_0_ decreased by 84.81% (F = 98.181, df = 117.620, *P* = 0.000) and 78.95% (F = 4.728, df = 191.503, *P* = 0.000), *r* decreased by 58.88% (F = 34.796, df = 145.910, *P* = 0.000) and 49.24% (F = 13.577, df = 169.040, *P* = 0.000), and *λ* decreased by 22.76% (F = 42.607, df = 137.806, *P* = 0.000) and 19.42% (F = 43.867, df = 136.917, *P* = 0.000) compared with those in the control group, respectively (*P* < 0.05) (Table 3). Additionally, the *T* in the LC_50_ imidacloprid (F = 43.867, df = 136.917, *P* = 0.000) and thiamethoxam (F = 6.873, df = 181.206, *P* = 0.000) -treatment groups was 0.16- and 0.12-fold higher than that of the control group, respectively. By contrast, the *T* in the LC_30_ thiamethoxam group (F = 0.118, df = 198, *P* = 0.000) significantly decreased by 5.98% relative to that in the control group (*P <* 0.05), whereas that in the LC_30_ imidacloprid group (F = 0.638, df = 198, *P* = 0.000) showed no significant change (*P >* 0.05) (Table 3).

**Table 3:** Mean value (± SE) of fertility parameters of *A*. *glycines* exposed to imidacloprid and thiamethoxam.

Population parameters	Control	LC_30_		LC_50_	
		Imidacloprid	Thiamethoxam	Imidacloprid	Thiamethoxam
Intrinsic rate of increase (*r*) (d^−1^)	0.4387 ± 0.0080a	0.3100 ± 0.0139b	0.3407 ± 0.0149b	0.1804 ± 0.0195c	0.2227 ± 0.0159c
Finite rate of increase (*λ*) (d^−1^)	1.5507 ± 0.0124a	1.3635 ± 0.0189b	1.4059 ± 0.0209b	1.1977 ± 0.0232c	1.2495 ± 0.0198c
Net reproductive rate (*R*_0_)	36.54 ± 2.156a	11.92 ± 1.303b	13.82 ± 1.466b	5.55 ± 0.916c	7.69 ± 1.028c
Mean generation time (*T*) (d)	8.20 ± 0.076b	7.99 ± 0.107bc	7.71 ± 0.109c	9.50 ± 0.219a	9.16 ± 0.097a

Means (±SE) followed by different letters in the same row are significantly different as calculated using the paired bootstrap test at the *P* < 0.05 level. Leaves treated with distilled water were used as the control.

SE = standard error.

**Fig 1:**
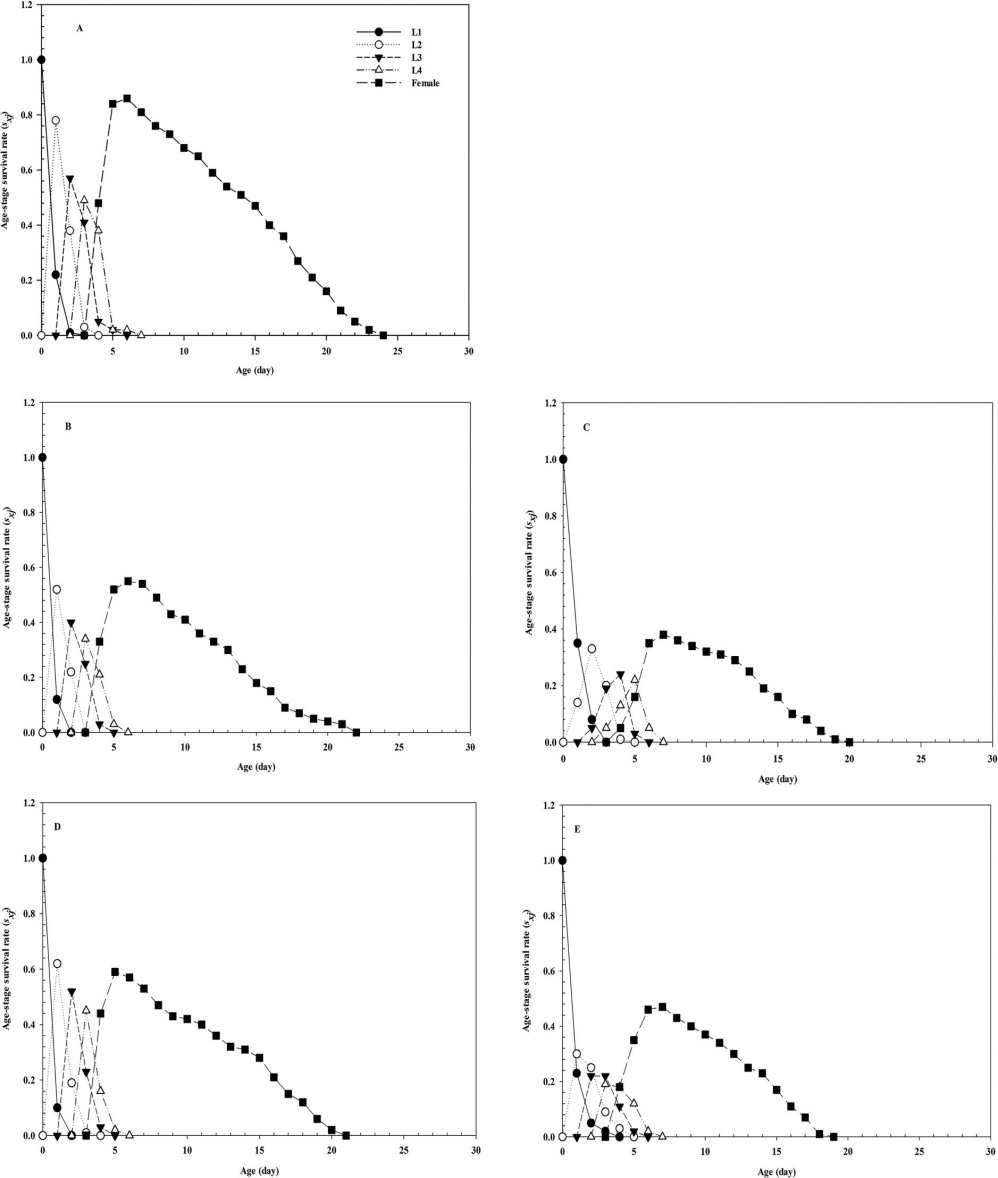
Age-stage specific survival rate (*s*_*xj*_) of *A*. *glycines*. The *s*_*xj*_ of *A*. *glycines* in the control (A), LC_30_ imidacloprid (B), LC_50_ imidacloprid (C), LC_30_ thiamethoxam (D), and LC_50_ thiamethoxam (E) groups. L1 = *s*_*xj*_ of the first instar nymphs; L2 = *s*_*xj*_ of the second instar nymphs; L3 = *s*_*xj*_ of the third instar nymphs; and L4 = *s*_*xj*_ of the fourth instar nymphs.

**Fig 2:**
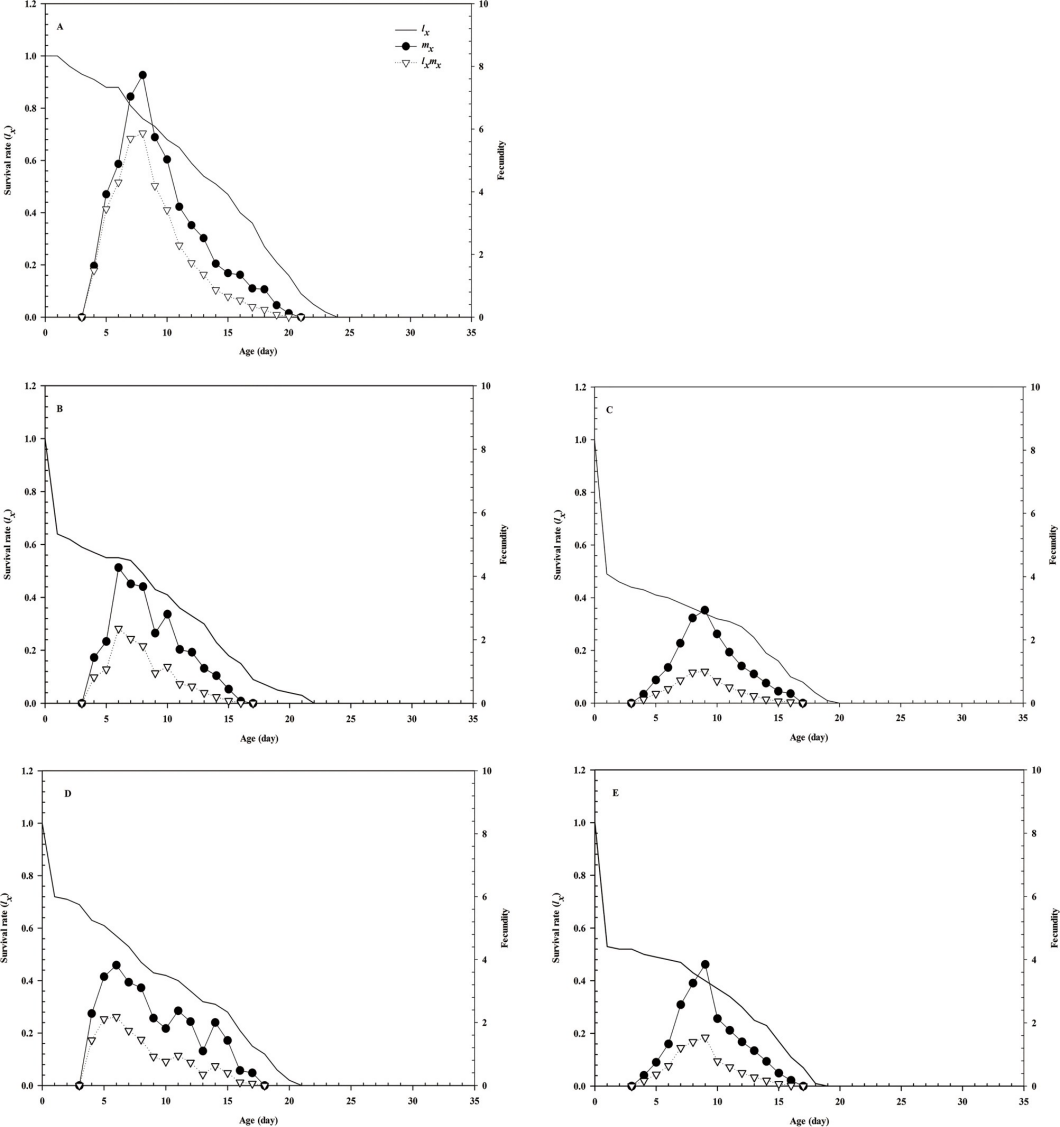
Age-specific survival rate (*l*_*x*_), fecundity (*m*_*x*_), and maternity (*l*_*x*_*m*_*x*_) of *A*. *glycines*. The *l*_*x*_, *m*_*x*_, and *l*_*x*_*m*_*x*_ of *A*. *glycines* in the control (A), LC_30_ imidacloprid (B), LC_50_ imidacloprid (C), LC_30_ thiamethoxam (D), and LC_50_ thiamethoxam (E) groups.

**Fig 3:**
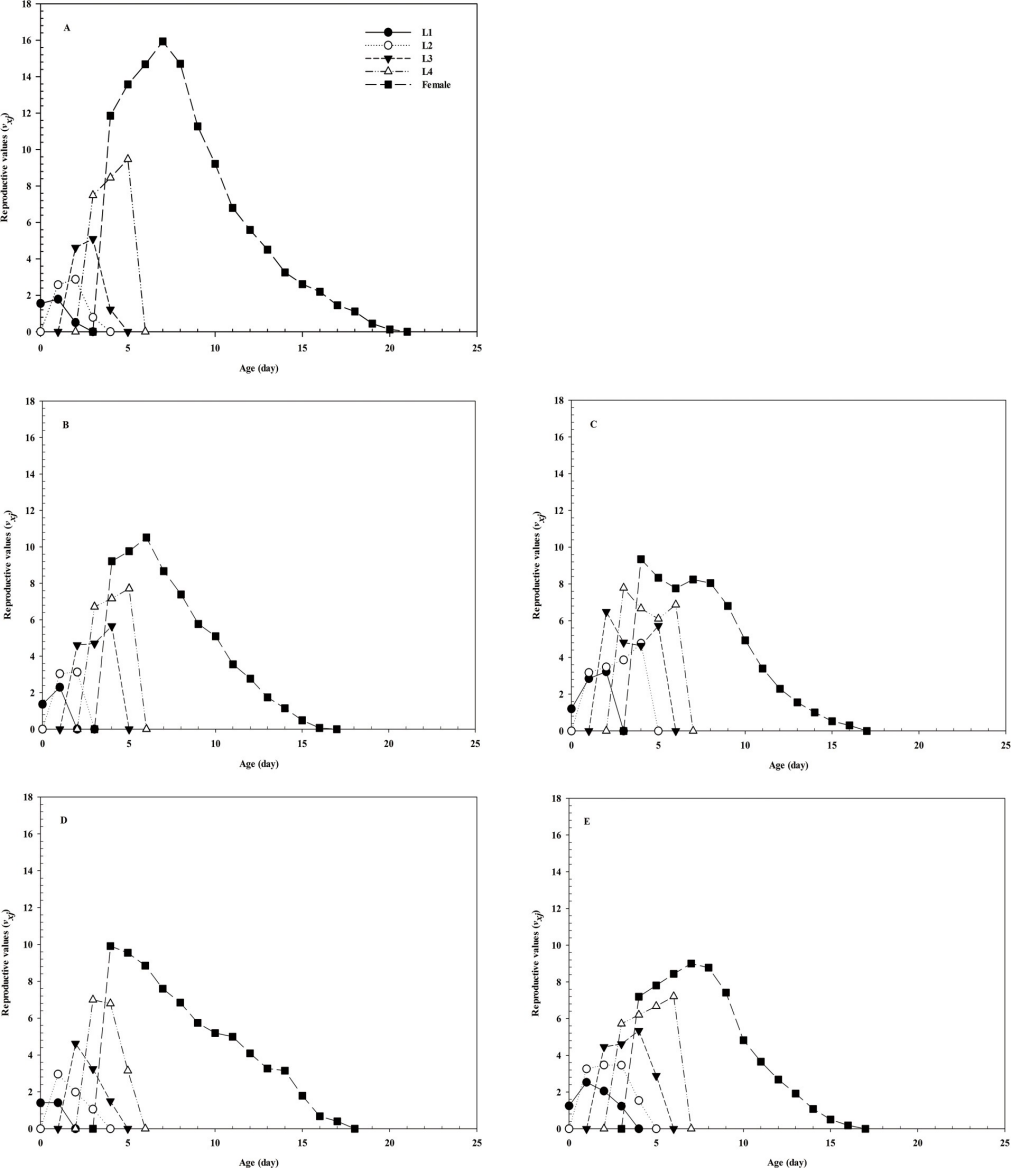
Age-stage-specific reproductive values (*v*_*xj*_) of *A*. *glycines*. The *v*_*xj*_ of *A*. *glycines* in the control (A), LC_30_ imidacloprid (B), LC_50_ imidacloprid (C), LC_30_ thiamethoxam (D), and LC_50_ thiamethoxam (E) groups. L1 = *v*_*xj*_ of first the instar nymphs; L2 = *v*_*xj*_ of the second instar nymphs; L3 = *v*_*xj*_ of the third instar nymphs; and L4 = *v*_*xj*_ of the fourth instar nymphs.

**Fig 4:**
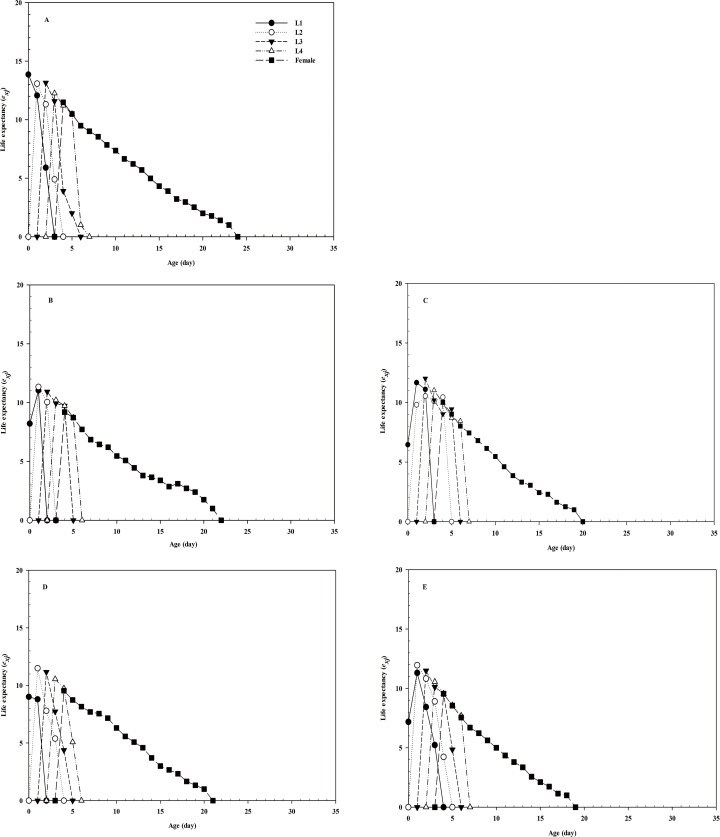
Life expectancy (*e*_*xj*_) of *A*. *glycines*. The *e*_*xj*_ of *A*. *glycines* in the control (A), LC_30_ imidacloprid (B), LC_50_ imidacloprid (C), LC_30_ thiamethoxam (D), and LC_50_ thiamethoxam (E) groups. L1 = *e*_*xj*_ of the first instar nymphs; L2 = *e*_*xj*_ of the second instar nymphs; L3 = *e*_*xj*_ of the third instar nymphs; and L4 = *e*_*xj*_ of the fourth instar nymphs.

Due to the different developmental rates of individuals, the age-stage-specific survival-rate curves showed obvious overlaps (**Fig 1**). The age-specific survival rate shows the probability that a first instar nymph will reach age *x*, and the curve of the age-specific survival rate is a simplified form of the curve of the age-stage survival rate, disregarding developmental stages. After treatment with imidacloprid and thiamethoxam, the *l*_*x*_ curve decreased significantly (**Fig 2**). The highest peak of *m*_*x*_ in the control group appeared on day 8 (**Fig 2**), whereas that in the LC_30_ imidacloprid group appeared on day 7 (1 day earlier than that in the control group). The highest peak of *m*_*x*_ in the LC_30_ thiamethoxam group appeared on day 6 (2 days earlier than that in the control group). Notably, the highest peak of *m*_*x*_ in the LC_50_ imidacloprid group appeared on day 10 (2 days later than that in the control group), whereas the highest peak of *m*_*x*_ in the LC_50_ thiamethoxam group appeared on day 9 (1 day later than that in the control group) (**Fig 2**). Furthermore, the values of age-specific maternity (*l*_*x*_*m*_*x*_) were significantly dependent on the *l*_*x*_ and *m*_*x*_, and the maximum *l*_*x*_*m*_*x*_ values were 8, 8, 9, 7, and 6 days for the control, LC_50_ imidacloprid, LC_50_ thiamethoxam, LC_30_ imidacloprid, and LC_30_ thiamethoxam treatment groups, respectively. The female reproductive values in the LC_30_ imidacloprid (F = 0.095, df = 28, *P* = 0.860) and thiamethoxam (F = 0.379, df = 29, *P* = 0.830) -treatment groups were higher than those in the LC_50_ imidacloprid and thiamethoxam groups, respectively (**Fig 3**). The age-stage life-expectancy curve (*e*_*xj*_) is shown in **Fig 4**. In the curve, the highest peak values of the first to fourth instar nymphs and female adults were lower in the treatment groups than in the control group.

## Discussion

We found that imidacloprid and thiamethoxam at LC_50_ significantly increased the APNP and TPNP and significantly decreased the mean fecundity per female adult as compared with those of the control, whereas the LC_30_ imidacloprid and thiamethoxam groups presented significantly shorter APNPs than the control group. This might be because different concentrations of imidacloprid and thiamethoxam exert different hormesis on soybean aphids [31]. The results showed that low-concentration insecticide stimulated growth, and high-concentration insecticide inhibited reproduction. One explanation might be that the lethal or sublethal concentrations of insecticide caused the immediate death of pests with weak resistance, whereas physiology and behavior changes occured in survivors [32,33]. These results indicated that different concentrations insecticides led to different coping strategies of soybean aphids. In the present study, both imidacloprid and thiamethoxam at LC_50_ and LC_30_ decreased the biological fitness of the F0 generation, which agreed with a previous study showing that clothianidin and acetamiprid at low-lethal concentrations adversely affected the biological fitness of melon aphids (*A*. *gossypii*) [34]. Another study reported that sublethal concentrations of spinetoram significantly decreased the biological fitness of the F1 generation of *Plutella xylostella* (L.) (Lepidoptera: Plutellidae) [35]. These results suggest that sublethal concentrations of insecticides can affect the biological fitness of pest populations. In this study, we found that LC_30_ imidacloprid and thiamthoxam significantly shortened the APNP, which might be related to the adaptation of soybean aphids to stress from low-concentration insecticides. By shortening the generation time, soybean aphids entered the reproduction period earlier and produced progeny faster, enabling the population to continue under pressure [36].

The effect on reproduction by different concentrations of imidacloprid and thiamethoxam was also reflected in the timing of the reproductive peak. The reproductive peak associated with imidacloprid and thiamethoxam treatment arrived earlier in the LC_30_ groups and later in the LC_50_ groups relative to that observed in the control group of soybean aphids. Additionally, we observed that LC_30_ treatment moved the reproductive peak 1 day earlier in the imidacloprid-treatment group and 2 days earlier in thiamethoxam-treatment group relative to the control. By contrast, the peaks observed in LC_50_ treatment groups were 2 days later (imidacloprid) and 1 day later (thiamethoxam) than that of the control. So, it had different biological and ecological effects on soybean aphids which dealed with different concentrations of the same agent or different agents of the same concentration [36]. Previous studies suggested that this might be related to differential expression of reproduction-related proteins. Ge et al. [37] found that protein content in male accessory glands of brown planthopper (*Nilaparvata lugen*) treated with triazophos was significantly higher than that in untreated controls, and that could be transmitted to female planthoppers during mating and then further promoted reproduction. Moreover, Zhou et al. [38] found that insecticide treatment significantly increases Vg content and promotes ovarian development, leading to mass reproduction of the white-backed planthopper (*Sogatella furcifera*) under triazophos stress. The findings of the present study offer insight into the mechanisms associated with the effects of low-lethal concentrations of insecticides on the reproduction of soybean aphids.

In the field, soybean aphids are usually exposed to insecticides at low-lethal or sublethal concentrations [39]. Subsequent generations of soybean aphids have developed low-level resistance to insecticides through the accumulation of mutations, leading to gradual increases in the extent of their resistance and eventual resurgence of soybean aphid populations [40]. Additionally, transgenerational hormesis can also lead to pest resurgence [11,41]. A previous study demonstrated that this process could lead to a significant increase in multigenerational reproduction of *Myzus persicae* following prolonged exposure to sublethal concentrations of imidacloprid in a greenhouse [36]. Therefore, these findings suggested the necessity to alternate insecticides with different modes of action in order to delay the development of resistance and transgenerational hormesis.

## Conclusion

The results showed that the biological fitness of soybean aphids was reduced following treatment with imidacloprid and thiamthoxam at LC_30_ and LC_50_. Moreover, low-concentration insecticide stress reduced the generation time and pre-reproductive periods while promoting the reproduction of progeny. These results support the view that insecticides at low-lethal or sublethal concentrations exert hormesis effects on pest populations. Furthermore, these findings demonstrated that the reproductive peak was advanced under stress associated with low-lethal concentrations of insecticides, which provided insight into the occurrence period of soybean aphids. Our future work will focus on determining the effects of imidacloprid and thiamethoxam on the physiology and behavior of soybean aphids after multiple generations of insecticide stress in an effort to provide a reference for the improved use of neonicotinoids.

## Supporting information

S1 Fig Age-stage-specific survival rate (*s*_*xj*_) of *A*. *glycines*. The *s*_*xj*_ of *A*. *glycines* in the control (A), LC_30_ imidacloprid (B), LC_50_ imidacloprid (C), LC_30_ thiamethoxam (D), and LC_50_ thiamethoxam (E) groups. L1 = *s*_*xj*_ of the first instar nymphs; L2 = *s*_*xj*_ of the second instar nymphs; L3 = *s*_*xj*_ of the third instar nymphs; and L4 = *s*_*xj*_ of the fourth instar nymphs.
(XLS)

S2 Fig Age-specific survival rate (*l*_*x*_), fecundity (*m*_*x*_), and maternity (*l*_*x*_*m*_*x*_) of *A*. *glycines*. The *l*_*x*_, *m*_*x*_, and *l*_*x*_*m*_*x*_ of *A*. *glycines* in the control (A), LC_30_ imidacloprid (B), LC_50_ imidacloprid (C), LC_30_ thiamethoxam (D), and LC_50_ thiamethoxam (E) groups.
(XLS)

S3 Fig Age-stage-specific reproductive values (*v*_*xj*_) of *A*. *glycines*. The *v*_*xj*_ of *A*. *glycines* in the control (A), LC_30_ imidacloprid (B), LC_50_ imidacloprid (C), LC_30_ thiamethoxam (D), and LC_50_ thiamethoxam (E) groups. L1 = *v*_*xj*_ of first the instar nymphs; L2 = *v*_*xj*_ of the second instar nymphs; L3 = *v*_*xj*_ of the third instar nymphs; and L4 = *v*_*xj*_ of the fourth instar nymphs.
(XLS)

S4 Fig Life expectancy (*e*_*xj*_) of *A*. *glycines*. The *e*_*xj*_ of *A*. *glycines* in the control (A), LC_30_ imidacloprid (B), LC_50_ imidacloprid (C), LC_30_ thiamethoxam (D), and LC_50_ thiamethoxam (E) groups. L1 = *e*_*xj*_ of the first instar nymphs; L2 = *e*_*xj*_ of the second instar nymphs; L3 = *e*_*xj*_ of the third instar nymphs; and L4 = *e*_*xj*_ of the fourth instar nymphs.
(XLS)

S1 Table Toxic effects of imidacloprid and thiamethoxam on the first instar nymphs of *A*. *glycines*. SE = standard error.
(XLS)

S2 Table Mean value (± SE) of life history parameters of *A*. *glycines* exposed to imidacloprid and thiamethoxam. Means (±SE) followed by different letters in the same row are significantly different as calculated using the paired bootstrap test at the *P* < 0.05 level. Leaves treated with distilled water were used as the control. SE = standard error; L1 = mean longevity of the first instar nymphs; L2 = mean longevity of the second instar nymphs; L3 = mean longevity of the third instar nymphs; L4 = mean longevity of the fourth instar nymphs; Fecundity = mean fecundity per female adult.
(DOC)

S3 Table Mean value (± SE) of fertility parameters of *A*. *glycines* exposed to imidacloprid and thiamethoxam. Means (±SE) followed by different letters in the same row are significantly different as calculated using the paired bootstrap test at the *P* < 0.05 level. Leaves treated with distilled water were used as the control. SE = standard error.
(DOC)
