# Supplementary material for: Effects of imidacloprid and thiamethoxam on the development and reproduction of the soybean aphid Aphis glycines
Source: PLoS One. 2021 Sep 16;16(9):e0250311. doi: 10.1371/journal.pone.0250311 (PMC8445468; doi:10.1371/journal.pone.0250311)
Supplement: S2 Table — Means (±SE) followed by different letters in the same row are significantly different as calculated using the paired bootstrap test at the P < 0.05 level. Leaves treated with distilled water were used as the control. SE = standard error; L1 = mean longevity of the first instar nymphs; L2 = mean longevity of the second instar nymphs; L3 = mean longevity of the third instar nymphs; L4 = mean longevity of the fourth instar nymphs; Fecundity = mean fecundity per female adult. (DOC) [file pone.0250311.s006.doc]

Raw data of reproductive growth of soybean aphid population in laboratory control group

"Aphis "

"Zhang"

"2020"

100

2,5

F,N1,N2,N3,N4,Female

N,N1,N2,N3,N4,Unknown

N1,N4

1,F,1,1,1,1,20,4,3,3,6,10,3,7,2,4,3,1,4,2,0,5,2,2,0,0,0,-1

2,F,1,1,1,1,19,5,4,3,6,9,5,2,5,3,5,2,2,4,1,2,0,0,0,0,-1

3,F,1,2,1,1,17,0,3,6,10,5,6,4,4,3,2,2,5,2,1,1,0,0,-1

4,F,1,1,1,1,18,0,3,4,6,9,6,6,7,6,3,4,0,3,6,4,1,0,0,-1

5,F,1,1,1,1,12,4,5,5,6,10,6,6,4,3,3,0,0,-1

6,F,1,2,1,1,11,2,4,7,10,6,6,4,6,0,0,0,-1

7,F,1,1,1,1,13,5,4,6,11,7,6,6,4,3,3,0,0,0,-1

8,F,1,1,1,1,12,6,7,5,6,11,8,4,2,3,4,0,0,-1

9,F,1,1,2,1,7,0,7,7,7,6,0,1,-1

10,F,2,1,1,1,2,5,6,-1

11,N,1,1,4,-1

12,N,2,2,-1

13,F,2,1,1,1,7,5,7,5,9,6,0,0,-1

14,F,1,1,1,1,8,5,5,7,6,9,6,1,0,-1

15,F,2,1,1,1,2,5,0,-1

16,F,1,1,2,1,4,4,0,12,0,-1

17,F,1,1,1,1,5,5,6,6,12,0,-1

18,F,2,1,1,1,12,3,2,7,7,6,6,4,3,6,2,0,0,-1

19,N,3,1,-1

20,F,1,1,1,1,14,1,5,6,8,7,6,9,2,5,3,2,2,0,0,-1

21,F,1,1,1,1,14,2,6,7,8,9,6,7,4,6,3,2,2,0,0,-1

22,F,1,1,2,1,11,1,7,8,7,6,6,4,6,3,0,0,-1

23,F,1,1,2,1,3,2,5,4,-1

24,N,1,1,2,-1

25,F,1,1,1,1,7,0,3,7,7,7,6,0,-1

26,N,2,-1

27,F,1,2,2,1,2,3,8,-1

28,F,1,1,1,1,15,0,2,4,9,8,6,6,5,0,4,0,2,3,0,0,-1

29,F,1,2,1,1,11,4,2,7,5,6,6,4,0,2,0,0,-1

30,F,1,1,2,1,15,6,3,6,8,6,8,3,4,3,2,3,2,0,0,0,-1

31,F,1,1,1,1,16,2,5,8,5,7,6,2,4,0,3,4,2,2,0,0,0,-1

32,F,1,1,1,1,16,3,4,7,7,7,4,8,5,3,4,3,1,2,0,0,0,-1

33,F,1,2,1,1,14,2,5,8,5,8,4,4,5,3,3,4,1,0,0,-1

34,F,1,1,1,1,15,1,3,5,9,8,4,8,3,4,2,3,0,1,0,0,-1

35,F,1,1,1,1,17,2,5,6,5,6,6,7,6,0,4,0,2,2,3,0,0,0,-1

36,F,1,1,1,1,19,0,8,4,5,8,7,7,4,6,0,4,0,3,2,3,2,0,0,0,-1

37,F,1,1,1,1,19,3,6,5,8,9,5,7,4,4,2,4,1,2,3,0,1,0,0,0,-1

38,F,1,1,1,1,11,6,6,4,7,10,6,7,5,3,0,0,-1

39,F,1,2,1,1,10,3,7,7,9,6,6,4,3,0,0,-1

40,F,1,2,1,1,9,5,3,7,10,5,6,4,4,0,-1

41,F,1,1,1,1,10,4,6,4,7,9,7,6,4,2,0,-1

42,N,2,2,2,-1

43,F,1,1,1,1,4,0,6,3,10,-1

44,F,1,2,1,1,5,4,5,7,9,0,-1

45,F,1,1,1,1,6,2,2,3,5,7,0,-1

46,F,1,2,1,1,16,5,7,8,8,6,4,3,3,2,2,2,2,3,0,0,0,-1

47,F,1,1,1,1,17,5,3,7,6,9,6,4,4,3,3,1,3,2,2,4,0,0,-1

48,F,1,1,2,2,13,3,6,7,9,5,4,4,2,2,2,1,0,0,-1

49,F,1,1,1,1,15,6,5,7,7,8,6,6,4,3,3,2,2,0,0,0,-1

50,F,1,1,1,1,11,3,7,7,7,9,6,4,2,0,0,0,-1

51,F,1,1,1,1,11,0,4,8,7,5,6,5,3,4,0,0,-1

52,F,1,1,1,1,9,0,6,5,5,8,6,1,0,0,-1

53,F,1,1,1,1,9,4,5,8,7,7,5,6,1,0,-1

54,N,2,1,-1

55,N,1,1,1,-1

56,F,1,2,1,1,2,2,3,-1

57,F,1,1,1,1,18,3,5,6,7,9,6,7,3,0,2,2,3,2,0,1,0,0,0,-1

58,F,1,1,1,1,18,4,5,5,8,5,4,5,3,2,5,4,1,1,2,2,0,0,0,-1

59,F,1,2,1,1,12,0,4,6,7,6,6,4,3,3,0,0,0,-1

60,F,1,1,1,1,16,0,4,5,7,9,6,6,4,4,3,4,2,3,0,0,0,-1

61,F,2,1,1,1,9,5,4,7,10,6,6,4,3,0,-1

62,F,1,1,1,1,9,0,5,7,7,5,6,4,5,3,-1

63,F,1,1,1,1,7,6,5,6,7,7,6,0,-1

64,F,2,1,1,1,6,4,3,7,7,6,0,-1

65,F,1,2,1,1,5,0,4,9,8,3,-1

66,F,1,1,1,1,6,5,7,5,7,6,9,-1

67,F,2,1,1,1,14,3,6,7,8,3,7,4,2,4,2,2,0,0,0,-1

68,F,2,1,1,1,13,4,7,5,6,6,5,4,0,3,2,2,0,0,-1

69,F,1,1,1,1,14,5,7,6,6,8,4,6,8,2,2,0,2,0,0,-1

70,F,1,1,1,1,14,7,5,6,7,9,8,6,0,4,4,4,2,0,0,-1

71,F,2,1,1,1,15,2,6,7,9,8,6,4,6,2,0,2,1,0,0,0,-1

72,F,1,2,1,1,5,5,3,8,0,0,-1

73,F,2,1,1,1,3,5,4,9,-1

74,F,2,1,1,1,3,6,0,9,-1

75,N,2,-1

76,N,2,-1

77,F,2,1,1,1,13,4,4,7,11,6,6,4,3,3,2,1,0,0,-1

78,F,1,2,1,1,13,0,3,7,9,6,6,4,3,3,2,2,0,0,-1

79,F,1,2,1,1,2,0,3,-1

80,F,1,2,1,1,2,0,8,-1

81,F,1,1,1,1,14,4,5,4,5,8,8,3,6,0,3,0,2,0,0,-1

82,F,1,1,1,1,14,3,6,5,8,10,4,6,4,4,3,6,2,0,0,-1

83,N,1,-1

84,F,1,1,1,1,20,4,3,5,6,11,7,5,6,2,3,3,0,2,2,2,1,0,0,0,0,-1

85,F,1,1,1,1,17,3,6,6,9,12,4,6,4,4,3,2,3,0,2,0,0,0,-1

86,F,2,1,1,1,16,4,7,7,5,8,7,2,3,4,4,2,3,2,0,0,0,-1

87,F,1,2,1,1,4,0,5,3,0,-1

88,N,1,-1

89,N,1,-1

90,F,1,1,1,1,17,4,6,6,8,9,7,6,4,4,2,3,0,2,2,0,0,0,-1

91,F,1,1,1,1,8,3,4,5,7,10,3,2,1,-1

92,F,2,1,1,1,7,5,6,6,9,6,2,1,-1

93,F,1,1,1,1,12,4,4,6,7,9,6,3,6,0,3,0,0,-1

94,F,1,1,1,1,12,2,6,6,7,8,6,9,5,6,0,0,0,-1

95,N,1,-1

96,F,2,1,1,1,8,5,4,6,9,6,4,3,0,-1

97,F,1,1,1,1,13,4,3,5,8,8,7,4,4,4,3,0,0,0,-1

98,F,1,1,1,1,9,3,4,5,7,8,8,8,0,0,-1

99,F,1,1,1,1,17,5,5,9,6,9,6,6,4,4,2,2,2,3,1,0,0,0,-1

100,F,1,1,1,1,8,2,6,5,8,5,9,0,1,-1

Raw data of reproductive growth of soybean aphid treated with LC30 imidacloprid

"Aphis "

"Zhang"

"2020"

100

2,5

F,N1,N2,N3,N4,Female

N,N1,N2,N3,N4,Unknown

N1,N4

1,F,1,1,1,1,18,2,0,4,6,4,2,3,0,2,2,0,2,0,0,0,0,0,0,-1

2,F,2,1,1,1,12,1,5,8,4,3,2,3,1,3,0,0,0,-1

3,F,1,2,2,1,11,5,4,6,3,3,2,3,0,0,0,0,-1

4,N,2,1,-1

5,F,1,1,1,1,4,2,0,0,0,-1

6,F,1,1,1,1,3,3,0,0,-1

7,N,-1

8,N,-1

9,F,1,1,1,1,4,1,6,0,0,-1

10,N,1,-1

11,F,1,1,1,1,9,5,3,4,6,2,3,6,0,0,-1

12,F,2,1,1,1,7,3,4,5,0,0,4,0,-1

13,F,1,1,1,1,7,4,2,5,5,2,0,4,-1

14,F,1,1,1,1,6,2,0,4,5,0,0,-1

15,F,2,1,1,1,17,2,6,4,5,3,3,2,2,2,2,2,1,0,0,0,0,0,-1

16,F,1,1,1,1,13,3,4,2,4,5,4,3,2,2,4,0,0,0,-1

17,F,1,2,1,1,13,1,5,4,6,3,3,2,2,2,0,0,0,0,-1

18,F,1,1,1,1,1,0,-1

19,N,-1

20,N,-1

21,N,-1

22,N,-1

23,N,-1

24,N,-1

25,F,1,1,1,1,1,0,-1

26,F,1,2,1,1,6,2,5,4,0,0,0,-1

27,F,1,2,1,1,6,3,4,6,0,0,0,-1

28,F,1,1,2,1,8,1,6,2,0,0,0,0,0,-1

29,N,2,1,-1

30,F,1,1,1,1,10,1,4,6,4,4,3,0,1,0,0,-1

31,F,2,1,1,1,9,2,5,4,3,3,0,3,0,0,-1

32,F,1,1,1,1,5,4,2,4,2,0,-1

33,F,1,1,1,1,5,0,3,5,5,0,-1

34,N,-1

35,N,-1

36,N,-1

37,F,1,1,1,1,18,3,2,5,6,2,4,3,4,2,2,4,1,0,0,0,0,0,0,-1

38,F,1,1,2,1,16,2,0,7,6,2,3,2,2,2,1,1,0,0,0,0,0,-1

39,F,1,2,1,1,15,4,4,5,6,4,3,2,2,2,3,2,0,0,0,0,-1

40,F,1,2,1,1,14,4,3,6,6,3,3,2,2,2,2,0,0,0,0,-1

41,F,1,1,1,1,5,4,2,7,4,0,-1

42,F,2,1,2,1,4,3,5,0,0,-1

43,N,-1

44,N,-1

45,N,-1

46,N,-1

47,N,-1

48,N,-1

49,F,1,1,1,1,4,2,2,0,4,-1

50,F,1,1,1,1,4,4,2,0,3,-1

51,N,2,-1

52,N,-1

53,N,-1

54,N,-1

55,F,1,2,1,1,4,2,5,4,0,-1

56,F,1,1,1,1,5,2,1,6,4,0,-1

57,F,1,1,2,1,10,1,5,4,6,3,2,3,2,0,0,-1

58,F,1,1,1,1,12,3,3,6,2,6,3,3,2,2,0,0,0,-1

59,F,1,1,1,1,9,2,4,5,4,3,2,4,0,0,-1

60,F,2,1,1,1,10,0,5,4,3,3,3,2,2,0,0,-1

61,F,1,1,1,1,10,2,2,5,4,3,3,3,2,2,0,-1

62,F,1,1,1,1,13,1,4,6,4,4,1,3,2,2,0,0,0,0,-1

63,F,1,1,2,1,9,2,5,4,6,4,3,0,2,0,-1

64,N,1,1,-1

65,F,2,1,1,1,4,2,0,4,0,-1

66,N,-1

67,N,-1

68,N,-1

69,N,-1

70,N,-1

71,F,1,1,1,1,13,3,0,4,4,8,5,3,2,2,5,0,0,0,-1

72,F,1,1,1,2,7,1,6,2,4,0,4,0,-1

73,F,1,1,1,1,8,4,2,5,5,0,0,4,0,-1

74,F,1,1,1,1,12,3,2,5,4,5,3,3,2,2,3,0,0,-1

75,F,1,1,1,1,14,2,2,6,3,6,5,3,2,0,2,6,0,0,0,-1

76,F,1,2,1,1,9,4,4,5,2,3,3,4,2,0,-1

77,F,1,1,2,1,9,2,5,3,7,2,3,0,3,0,-1

78,F,1,1,1,1,11,3,0,6,4,6,3,3,4,0,0,0,-1

79,F,1,1,1,1,10,4,0,4,4,2,3,0,3,0,0,-1

80,N,-1

81,N,-1

82,N,-1

83,N,-1

84,N,1,-1

85,F,1,1,1,1,7,3,1,6,2,3,0,5,-1

86,N,-1

87,F,1,2,1,1,10,4,5,6,4,3,5,0,2,0,0,-1

88,F,1,1,1,1,12,4,2,4,6,4,3,3,2,2,0,0,0,-1

89,F,1,2,2,1,9,5,6,4,3,3,2,2,0,0,-1

90,F,1,1,1,1,15,3,2,4,6,5,1,3,2,4,2,2,0,0,0,0,-1

91,F,2,1,1,1,12,2,4,8,7,2,3,2,2,0,0,0,0,-1

92,N,-1

93,N,-1

94,N,2,-1

95,F,1,1,1,1,4,2,2,0,0,-1

96,N,-1

97,N,-1

98,N,-1

99,N,-1

100,F,1,1,1,1,7,0,3,4,5,4,0,3,-1

Raw data of reproductive growth of soybean aphid treated with LC30 thiamethoxam

"Aphis "

"Zhang"

"2020"

100

2,5

F,N1,N2,N3,N4,Female

N,N1,N2,N3,N4,Unknown

N1,N4

1,F,1,1,2,1,15,4,3,3,5,5,3,2,0,4,0,0,0,3,0,0,-1

2,F,1,1,1,1,13,4,5,4,6,1,6,2,0,3,6,0,0,0,-1

3,N,-1

4,F,2,1,1,1,11,2,5,1,2,2,4,2,0,3,0,0,-1

5,F,1,1,1,1,15,4,4,6,3,5,3,3,1,0,5,4,0,6,0,0,-1

6,F,1,1,1,1,7,6,7,5,8,0,0,2,-1

7,N,2,-1

8,F,1,1,1,1,11,5,6,9,4,2,1,3,3,5,0,0,-1

9,F,1,2,1,1,1,0,-1

10,F,1,1,1,1,15,3,4,9,6,4,2,4,3,0,0,6,0,0,0,0,-1

11,N,-1

12,F,1,1,1,1,3,3,0,2,-1

13,F,1,1,1,1,5,2,3,7,0,3,-1

14,F,1,1,1,1,12,1,5,11,4,1,3,0,0,6,0,0,0,-1

15,F,1,1,1,1,9,1,7,3,4,2,1,3,2,0,-1

16,N,-1

17,N,-1

18,F,1,1,1,1,1,0,-1

19,N,-1

20,N,-1

21,N,-1

22,F,1,1,1,1,7,4,6,2,0,2,0,0,-1

23,F,1,1,1,1,3,2,0,6,-1

24,F,1,1,1,1,2,5,0,-1

25,F,1,1,2,1,9,4,7,2,4,2,0,1,0,6,-1

26,F,1,1,1,1,13,3,7,3,2,2,0,0,3,5,0,0,0,4,-1

27,N,2,1,-1

28,N,-1

29,N,-1

30,F,1,1,1,1,9,4,2,1,4,8,6,0,6,4,-1

31,F,1,1,2,1,3,2,9,0,-1

32,N,1,2,-1

33,N,1,-1

34,N,-1

35,F,1,1,1,1,5,3,7,2,3,0,-1

36,F,1,1,1,1,12,5,2,4,4,7,3,0,3,0,0,6,0,-1

37,F,1,1,1,1,4,2,0,2,3,-1

38,N,-1

39,F,2,1,1,1,7,3,5,1,2,0,0,0,-1

40,F,1,1,1,1,13,4,4,6,1,7,3,1,3,0,0,5,0,0,-1

41,N,-1

42,F,1,1,1,1,11,2,8,1,2,6,2,2,4,6,0,0,-1

43,F,1,1,1,1,8,4,0,2,0,2,6,3,5,-1

44,N,1,2,-1

45,F,1,1,1,1,2,2,0,-1

46,N,-1

47,N,1,1,3,-1

48,F,1,2,1,1,7,3,10,7,0,5,0,0,-1

49,F,1,1,2,1,4,5,4,1,0,-1

50,F,1,1,1,1,12,3,2,0,9,7,0,2,5,6,0,0,0,-1

51,F,1,1,1,1,4,0,3,1,1,-1

52,N,-1

53,N,-1

54,F,1,1,1,1,12,3,4,2,7,3,3,5,2,7,0,0,0,-1

55,F,1,1,1,1,4,5,4,4,0,-1

56,N,-1

57,F,1,1,1,1,9,3,5,2,4,1,3,3,0,6,-1

58,F,1,1,1,1,4,2,0,0,0,-1

59,N,1,2,-1

60,F,1,1,2,1,12,3,6,2,6,1,1,3,0,0,0,0,0,-1

61,F,2,1,1,1,2,0,0,-1

62,F,1,1,1,1,11,5,5,2,6,3,2,2,0,3,0,5,-1

63,F,1,1,1,1,12,4,6,3,3,4,4,0,5,6,0,0,0,-1

64,N,1,2,-1

65,F,2,1,1,1,3,1,3,2,-1

66,F,1,1,2,1,14,1,4,8,4,0,0,5,0,0,4,0,0,0,0,-1

67,N,-1

68,F,1,1,1,1,17,2,6,0,4,6,5,4,3,4,0,0,5,0,0,0,0,0,-1

69,N,-1

70,F,1,1,1,1,5,4,1,6,3,0,-1

71,F,1,2,1,1,11,2,4,0,2,0,2,1,6,0,0,0,-1

72,N,-1

73,F,2,1,2,1,1,3,-1

74,F,1,1,1,1,15,5,7,7,4,1,0,2,2,0,0,3,0,0,0,0,-1

75,F,1,1,1,1,14,3,5,7,5,6,4,2,0,1,0,0,4,0,0,-1

76,N,2,2,-1

77,F,1,1,1,1,16,4,5,1,7,4,1,1,5,0,0,0,0,0,3,0,0,-1

78,N,-1

79,F,1,1,1,1,6,5,5,4,2,0,2,-1

80,F,1,1,1,1,17,4,3,6,5,2,0,0,3,0,2,3,0,0,0,0,0,0,-1

81,F,1,1,1,1,13,2,5,2,4,4,1,2,2,0,0,4,0,0,-1

82,N,1,2,-1

83,F,1,1,1,1,16,3,6,2,4,3,1,2,4,0,0,0,6,0,0,0,0,-1

84,N,-1

85,F,1,1,1,1,15,3,2,2,0,4,1,4,0,2,0,1,4,0,0,0,-1

86,F,1,1,1,1,8,5,6,1,4,0,4,3,0,-1

87,N,-1

88,F,1,1,1,1,16,3,4,4,1,1,0,2,4,3,0,5,4,0,0,0,0,-1

89,N,-1

90,F,1,1,1,1,13,5,6,2,9,2,2,4,3,0,0,6,5,0,-1

91,N,2,-1

92,N,-1

93,N,-1

94,F,1,2,1,1,14,2,1,2,7,4,0,2,0,4,6,0,0,0,0,-1

95,N,-1

96,F,1,1,1,2,13,5,2,2,6,2,2,1,0,0,4,5,0,0,-1

97,F,2,1,1,1,8,3,6,1,4,1,0,3,0,-1

98,N,-1

99,N,-1

100,F,1,1,1,1,14,2,4,3,6,1,1,3,4,0,5,0,7,0,0,-1

Raw data of reproductive growth of soybean aphid treated with LC50 imidacloprid

"Aphis "

"Zhang"

"2020"

100

2,5

F,N1,N2,N3,N4,Female

N,N1,N2,N3,N4,Unknown

N1,N4

1,N,2,1,-1

2,F,2,1,1,2,14,0,0,4,2,5,2,2,0,0,1,0,0,0,0,-1

3,F,1,1,1,1,15,6,3,4,4,3,7,7,0,2,2,0,2,0,0,0,-1

4,F,3,1,1,1,2,2,0,-1

5,F,3,1,1,1,2,2,0,-1

6,N,2,-1

7,N,1,-1

8,N,1,-1

9,N,2,-1

10,N,1,-1

11,N,-1

12,F,2,2,1,2,6,0,4,0,0,0,0,-1

13,F,3,1,1,1,6,0,0,2,0,0,2,-1

14,F,2,1,1,1,12,0,0,0,3,2,3,2,0,1,0,2,0,-1

15,F,2,1,1,1,12,5,0,4,3,6,3,2,2,1,0,0,0,-1

16,F,2,1,1,1,11,0,0,3,4,4,4,2,2,3,1,0,-1

17,F,2,1,1,1,10,4,0,2,2,6,3,5,0,3,1,-1

18,N,-1

19,N,-1

20,N,-1

21,N,-1

22,N,-1

23,N,-1

24,N,-1

25,N,-1

26,F,1,2,2,1,4,7,4,0,0,-1

27,N,-1

28,N,-1

29,N,-1

30,F,2,1,1,1,10,0,1,2,4,2,3,4,4,3,1,-1

31,F,1,2,2,2,9,0,4,4,4,2,0,0,0,0,-1

32,F,2,2,1,1,9,0,0,5,4,4,5,0,0,2,-1

33,F,3,1,2,1,7,0,0,4,3,0,0,1,-1

34,F,2,2,2,1,7,6,3,4,5,0,0,0,-1

35,F,1,1,1,1,15,6,4,5,3,2,4,3,4,2,1,2,0,0,0,0,-1

36,F,3,1,1,1,7,0,4,2,0,0,0,1,-1

37,F,2,2,1,1,7,0,0,4,0,0,0,2,-1

38,N,-1

39,F,2,2,1,1,1,0,-1

40,N,-1

41,N,-1

42,N,-1

43,N,-1

44,N,-1

45,N,-1

46,N,-1

47,N,2,2,1,-1

48,N,-1

49,N,-1

50,N,-1

51,F,3,1,1,1,4,0,2,0,0,-1

52,F,2,2,1,1,3,3,2,0,-1

53,N,-1

54,N,-1

55,N,-1

56,N,-1

57,N,-1

58,N,-1

59,F,2,2,1,1,1,0,-1

60,F,1,1,1,1,14,0,8,1,0,3,4,3,2,2,0,0,0,0,0,-1

61,F,2,1,1,1,13,0,0,6,3,4,3,2,1,1,2,0,0,0,-1

62,F,2,1,2,1,6,0,0,3,0,0,0,-1

63,F,1,1,1,1,14,0,0,0,2,3,4,3,2,2,2,0,0,2,0,-1

64,F,1,2,1,1,13,0,0,2,3,4,3,2,3,1,0,0,1,0,-1

65,N,-1

66,N,-1

67,N,2,2,-1

68,N,-1

69,N,-1

70,F,3,1,1,1,8,6,3,3,4,5,0,0,0,-1

71,F,2,2,1,1,8,0,0,3,4,0,0,2,0,-1

72,F,1,1,1,1,15,0,2,0,2,3,7,3,2,2,1,0,1,0,0,0,-1

73,N,-1

74,N,-1

75,N,-1

76,N,-1

77,N,-1

78,N,-1

79,N,-1

80,N,-1

81,N,-1

82,N,2,2,-1

83,F,1,2,2,1,5,0,2,4,0,0,-1

84,N,-1

85,N,-1

86,N,-1

87,N,-1

88,N,-1

89,F,2,1,1,1,4,0,2,3,0,-1

90,F,2,1,1,1,11,0,1,2,3,4,5,2,2,0,0,0,-1

91,F,2,2,1,1,10,0,2,3,4,6,2,2,1,1,0,-1

92,F,1,2,1,1,11,0,0,2,3,3,5,2,0,0,1,0,-1

93,N,-1

94,N,-1

95,F,1,2,1,1,11,4,0,1,2,4,2,2,0,1,1,0,-1

96,F,2,1,1,2,8,0,0,4,1,5,0,0,1,-1

97,F,2,1,2,1,8,1,2,2,0,4,0,0,0,-1

98,N,-1

99,N,-1

100,F,3,2,1,1,6,0,3,0,0,0,1,-1

Raw data of reproductive growth of soybean aphid treated with LC50 thiamethoxam

"Aphis "

"Zhang"

"2020"

100

2,5

F,N1,N2,N3,N4,Female

N,N1,N2,N3,N4,Unknown

N1,N4

1,N,-1

2,N,-1

3,F,1,1,1,1,15,2,4,2,2,4,7,2,4,1,3,0,0,1,0,0,-1

4,N,2,1,-1

5,F,2,1,1,2,10,3,2,2,7,0,0,1,0,0,0,-1

6,F,1,3,1,2,11,2,2,5,2,4,1,2,2,0,1,0,-1

7,F,1,1,2,1,10,0,2,2,2,5,2,4,4,0,0,-1

8,N,3,2,-1

9,F,1,1,1,1,11,0,1,2,3,0,1,8,2,1,1,0,-1

10,F,1,1,1,1,12,0,3,2,2,3,7,4,4,0,0,0,2,-1

11,N,4,-1

12,F,1,1,1,2,11,4,5,2,3,8,4,2,5,0,0,0,-1

13,N,-1

14,N,-1

15,F,1,1,1,1,12,0,2,3,3,6,1,2,1,2,1,0,0,-1

16,N,-1

17,N,-1

18,N,-1

19,N,-1

20,F,1,1,1,1,11,0,0,5,2,3,7,4,3,0,0,0,-1

21,N,1,-1

22,F,1,1,1,1,11,0,0,7,2,0,6,3,1,0,1,1,-1

23,N,-1

24,N,-1

25,N,-1

26,F,2,2,1,1,9,4,5,3,6,4,2,0,0,1,-1

27,N,3,-1

28,F,2,1,1,1,8,0,0,0,1,4,0,2,2,-1

29,N,-1

30,N,-1

31,N,-1

32,N,-1

33,N,-1

34,F,1,1,1,1,11,0,1,6,2,4,3,1,2,0,0,0,-1

35,N,-1

36,N,-1

37,N,-1

38,F,1,1,1,1,9,0,0,0,4,6,4,1,2,0,-1

39,F,1,1,1,1,13,1,4,2,0,2,4,3,1,2,1,1,0,0,-1

40,F,2,2,1,1,4,0,5,3,0,-1

41,F,1,2,1,1,13,2,2,4,1,0,6,0,2,1,1,0,0,0,-1

42,F,2,1,2,1,12,4,3,4,3,3,1,1,2,2,1,0,0,-1

43,F,2,1,1,1,8,0,0,5,2,7,2,0,0,-1

44,F,1,2,1,1,8,0,0,3,0,5,0,3,1,-1

45,N,-1

46,F,1,1,1,1,14,4,4,0,2,7,5,2,3,2,4,2,2,0,0,-1

47,F,1,1,2,1,6,0,0,3,1,0,0,-1

48,F,1,1,1,1,13,2,2,3,4,4,4,2,1,4,0,3,0,0,-1

49,F,2,1,2,1,6,0,2,5,3,0,0,-1

50,F,1,1,1,1,10,2,0,0,5,4,4,3,1,0,2,-1

51,F,1,1,1,1,8,0,0,0,4,6,0,0,2,-1

52,N,-1

53,N,-1

54,F,1,1,1,1,14,2,0,3,0,2,2,0,2,3,2,4,2,0,0,-1

55,N,-1

56,N,-1

57,F,2,2,1,1,6,0,1,3,5,1,0,-1

58,F,2,1,2,1,8,0,1,4,2,0,1,0,0,-1

59,F,1,1,1,1,12,0,3,4,5,4,2,4,1,2,1,0,0,-1

60,F,2,1,1,1,7,0,0,3,6,3,0,0,-1

61,N,-1

62,N,-1

63,F,2,1,2,1,7,0,2,4,3,2,2,0,-1

64,N,-1

65,N,-1

66,N,-1

67,F,3,1,1,1,5,0,5,5,4,0,-1

68,N,-1

69,F,2,1,1,1,5,0,0,4,0,3,-1

70,F,1,1,1,1,6,0,0,0,3,4,4,-1

71,F,2,2,1,1,3,0,2,4,-1

72,F,1,1,1,1,14,4,2,1,3,3,4,4,2,2,1,0,0,0,0,-1

73,F,2,2,1,1,2,0,0,-1

74,F,2,1,1,1,2,0,0,-1

75,F,1,1,1,1,13,0,1,0,0,4,5,4,2,1,0,1,0,0,-1

76,F,4,1,1,1,1,0,-1

77,F,1,2,1,1,3,0,0,0,-1

78,F,1,1,2,1,4,0,0,5,0,-1

79,N,-1

80,N,-1

81,N,-1

82,N,-1

83,N,-1

84,N,-1

85,F,2,1,1,1,12,3,2,3,2,7,1,2,2,4,0,0,0,-1

86,N,-1

87,N,-1

88,N,-1

89,F,1,2,1,1,4,0,0,5,4,-1

90,F,1,2,1,1,3,0,0,0,-1

91,N,-1

92,N,-1

93,F,2,1,1,1,11,1,2,3,6,4,3,3,3,2,0,0,-1

94,F,1,2,1,1,6,0,0,3,7,0,2,-1

95,N,-1

96,N,-1

97,N,-1

98,N,-1

99,N,-1

100,N,-1
